# Comprehensive pan-cancer analysis of RPRD1B as a promising diagnostic and prognostic biomarker

**DOI:** 10.1007/s12672-025-03805-4

**Published:** 2026-01-06

**Authors:** Ye Tian, Xuning Wang, Rui Tang, Xiaojuan Wang

**Affiliations:** 1 Beijing Tsinghua Changgung Hospital, School of Clinical Medicine, Tsinghua Medicine, Tsinghua University, Beijing, China; 2 Institute for Organ Transplant and Bionic Medicine, Beijing Key Laboratory of Liver Transplantation and Bionic Manufacturing, Tsinghua University, Beijing, China; 3 Department of General Surgery, General Hospital of Northern Theater Command, Shenyang, China; 4 Hepato-Pancreato-Biliary Center, Beijing Tsinghua Changgung Hospital, Key Laboratory of Digital Intelligence Hepatology (Ministry of Education), School of Clinical Medicine, Tsinghua Medicine, Tsinghua University, Beijing, China

**Keywords:** RPRD1B (regulation of nuclear pre-mRNA domain containing 1B), Pan-cancer, Prognosis, Diagnosis, Biomarker

## Abstract

**Background:**

Regulation of nuclear pre-mRNA domain containing 1B (RPRD1B, also known as CREPT), has emerged as a potential factor in cancer development. Despite growing evidence, a comprehensive evaluation of RPRD1B across different cancer types has not been conducted. This study aims to explore its prognostic significance in pan-cancer analysis.

**Methods:**

Online databases and platforms such as The Cancer Genome Atlas (TCGA), Genotype Tissue Expression (GTEx), the Human Protein Atlas (HPA), cBioPortal, TIMER2.0, BioGRID, and SangerBox were used to gather data on RPRD1B across various cancers. The pan-cancer patient datasets were analyzed to explore the relationships among RPRD1B expression, genetic alterations, protein interactions, tumor immunity, and clinical features.

**Results:**

The findings indicate that RPRD1B was upregulated in most tumor types compared to normal tissues, and increased protein levels observed in several cancers. Notably, high RPRD1B expression was associated with the infiltration of immune cells, endothelial cells, and cancer-associated fibroblasts (CAFs), as well as the expression of tumor immune markers in certain cancers. Additionally, elevated RPRD1B expression proved useful for aiding cancer diagnosis and predicting prognosis across multiple cancer types. In vitro studies showed that RPRD1B was upregulated in colon adenocarcinoma (COAD) cell lines, where it promoted cell proliferation and invasion. In vivo experiments further demonstrated that knocking down RPRD1B significantly suppressed tumor growth.

**Conclusion:**

High RPRD1B expression contributes to cancer progression and may function as a key biomarker for the diagnosis and prognosis across various tumor types.

## Introduction

Cancer, as a major public health issue that poses a severe threat to human health and lives, has become a critical barrier to increasing life expectancy [1]. The occurrence of cancer is closely linked to various risk factors, including smoking, drinking, poor dietary habits, obesity, and lack of exercise [2]. While lifestyle interventions can reduce cancer risk [3], early screening and regular physical exams remain the most effective strategies for lowering cancer mortality, especially in high-risk populations [4–6].

Cancer pathogenesis is highly complex, and the significant heterogeneity across different cancer types underscores the need for innovative strategies to patient outcomes. Although current treatments like surgery, radiotherapy, chemotherapy, and targeted therapy have boosted survival rates [7], their effectiveness remains limited—particularly in advanced cancers, where prognosis remains poor. Furthermore, the difficulties in personalizing treatment and predicting individual responses underscore the urgent need for reliable biomarkers to improve prognostic assessment and refine treatment choices.

In recent years, research on biomarkers has increasingly become a vital tool for cancer diagnosis and treatment. Cancer biomarkers encompass a broad spectrum of biochemical entities, such as nucleic acids, proteins, sugars, small metabolites, cytogenetic and cellular dynamics parameters, as well as entire tumor cells found in body fluids [8]. These biomarkers can be used not only for early detection and screening but also for aiding the evaluation of disease progression and prognosis, aiding doctors in developing personalized treatment plans, which can enhance patient survival rates and quality of life.

Regulation of nuclear pre-mRNA domain containing 1B (RPRD1B, also known as CREPT) has become a gene of particular interest due to its link to tumorigenesis and disease progression [9]. Previous studies indicate that RPRD1B may be connected to various tumors. RPRD1B interacts with RNAPII to enhance transcription initiation and gene expression in tumor cells [10]. Additionally, RPRD1B promotes gene expression by affecting multiple signaling pathways, such as Wnt [11] and STAT3 [12]. Another study suggests RPRD1B can form a complex with Ku70 to participate in DNA damage repair [13]. These findings support further research into RPRD1B as a potential prognostic biomarker and provide a basis for exploring its roles across diverse malignancies.

Although previous studies have confirmed that RPRD1B has a pro-cancer effect in some tumors, the existing evidence still has limitations and does not systematically explain the molecular regulatory network of RPRD1B at the pan-cancer level or its potential as a biomarker. Therefore, a comprehensive investigation was conducted to explore the specific role of RPRD1B in human cancers, explored its value across pan-cancer, and provided new molecular markers and a theoretical basis for early diagnosis, personalized treatment, and prognosis assessment. This research is valuable for advancing precision cancer diagnosis and treatment.

## Methods and materials

### Gene expression analysis of RPRD1B

TIMER2.0 database (http://timer.comp-genomics.org/) [14], The Cancer Genome Atlas (TCGA), and Xiantao Academic (https://www.xiantaozi.com/) were used to examine differences in RPRD1B mRNA levels between various tumor tissues and corresponding normal tissues. For tumors lacking normal tissue counterparts, we analyzed the Genotype-Tissue Expression database (GTEx) and used GEPIA2 (Gene Expression Profiling Interactive Analysis 2, http://gepia2.cancer-pku.cn/) [15] to generate plots showing mRNA expression differences. Significant differences were identified using Wilcoxon tests. The abbreviations of the 33 tumors are listed in Table 1.

**Table 1:** Pan-cancers and the corresponding abbreviations Cancer Type Abbreviation

Cancer type	Abbreviation
Adrenocortical carcinoma	ACC
Bladder urothelial carcinoma	BLCA
Breast invasive carcinoma	BRCA
Cervical squamous cell carcinoma and endocervical adenocarcinoma	CESC
Cholangiocarcinoma	CHOL
Colon adenocarcinoma	COAD
Lymphoid neoplasm diffuse large b-cell lymphoma	DLBC
Esophageal carcinoma	ESCA
Glioblastoma multiforme	GBM
Head and neck squamous cell carcinoma	HNSC
Kidney chromophobe	KICH
Kidney renal clear cell carcinoma	KIRC
Kidney renal papillary cell carcinoma	KIRP
Acute myeloid leukemia	LAML
Brain lower grade glioma	LGG
Liver hepatocellular carcinoma	LIHC
Lung adenocarcinoma	LUAD
Lung squamous cell carcinoma	LUSC
Mesothelioma	MESO
Ovarian serous cystadenocarcinoma	OV
Pancreatic adenocarcinoma	PAAD
Pheochromocytoma and paraganglioma	PCPG
Prostate adenocarcinoma	PRAD
Rectum adenocarcinoma	READ
Sarcoma	SARC
Skin cutaneous melanoma	SKCM
Stomach adenocarcinoma	STAD
Testicular germ cell tumors	TGCT
Thyroid carcinoma	THCA
Thymoma	THYM
Uterine corpus endometrial carcinoma	UCEC
Uterine carcinosarcoma	UCS
Uveal melanoma	UVM

The expression and subcellular localization of RPRD1B were assessed through immunohistochemistry using the Human Protein Atlas (HPA) database (https://www.proteinatlas.org/) [16].

### Gene mutation analysis of RPRD1B

The online database cBioPortal (https://www.cbioportal.org/) was used to analyze the frequency of pan-cancer somatic mutations in RPRD1B [17, 18]. Genetic alterations included somatic mutation, amplification, and deep deletion. Additionally, the database provides information about mutation sites.

### Gene-related enrichment analysis of RPRD1B

BioGRID (https://thebiogrid.org/) is an online platform for exploring gene interactions and functions, as well as identifying co-expressed genes [19]. 246 genes that interact with RPRD1B were obtained through BioGRID, and visualization of these interactions was performed using Cytoscape [20]. Protein interactions were further analyzed using TIMER2.0 (http://timer.cistrome.org/). Gene Ontology (GO) and Kyoto Encyclopedia of Genes and Genomes (KEGG) functional enrichment analyses were conducted using Xiantao Academic (https://www.xiantaozi.com/).

### Analysis of RPRD1B and Immune infiltration

The potential link between RPRD1B expression levels and immune infiltration was examined using the TIMER2.0 database (http://timer.cistrome.org/) [21]. The relationships between RPRD1B expression and immune cells, cancer-associated fibroblasts (CAFs), and endothelial cells were analyzed across various cancer types. The algorithms used included EPIC, MCP-COUNTER, and TIDE.

### Correlation analysis between RPRD1B and tumor immunity markers

The relationships between RPRD1B expression and key tumor immunity biomarkers such as microsatellite instability (MSI), tumor mutation burden (TMB), neoantigens, and immune checkpoints (ICP) [22] across different tumors from TCGA cohorts were examined using the SangerBox website (http://sangerbox.com/home.html) [23].

### Prognostic analysis of RPRD1B

The prognostic significance of RPRD1B expression across 33 cancer types was assessed based on overall survival (OS) and progression-free interval (PFI). Study groups were divided into RPRD1B-high and RPRD1B-low subgroups based on the median RPRD1B expression value, with 50% cut-off high and 50% cut-off low. Survival outcomes were compared using Kaplan–Meier curve analysis with log-rank testing, supported by multivariate Cox proportional hazards regression to identify independent prognostic factors. Correlation analyses were conducted using the Xiantao Academic (https://www.xiantaozi.com/) online analysis tool. Prognostic indicators evaluated included hazard ratios (HR), 95% confidence intervals, and p-values, with *P* < 0.05 indicating statistical significance.

### Diagnostic efficacy of RPRD1B for different types of tumors

The TCGA database was used to investigate the diagnostic significance of RPRD1B across various tumor types. The relationship between pathological stage and RPRD1B expression was analyzed using R packages (ggplot2 [3.4.4], stats [4.2.1], car [3.1-0]). The R package “pROC” was then employed to calculate the AUC (Area Under Curve) values of RPRD1B at the pan-cancer level. Visualization was conducted using the R package ggplot2.

### Cell culture

HEK293T and SW480 cell lines were cultured in Dulbecco’s Modified Eagle’s Medium (DMEM). DLD1 was cultured in RPMI1640 medium, and SW620 was cultured in L-15 medium supplemented with 10% fetal bovine serum and penicillin/streptomycin. Media and serum were purchased from Invitrogen. All cells were maintained at 37 °C in a humidified atmosphere with 5% CO2. For generating RPRD1B stable knockdown cell lines, cells were infected with lentivirus and then selected using puromycin at different concentrations: 2 μg/ml (DLD-1), 1.5 μg/ml (SW480), and 2 μg/ml (SW620).

### Real-time quantitative PCR

qRT-PCR was performed using SuperReal PreMix Plus (SYBR Green) (TIANGEN, Beijing, China). The primers were as follows: RPRD1B: F: GGCAAGAACGAAGTGTGTATGG; R: CTGCCAGGGTAGTCGTCATC. β-actin: F: GTGTGTGACAATGGCTCTGG; R: TCCCAGTTGGTGATGATGCC. 2 − ΔΔCt values were calculated to perform the quantitative analysis.

### Western blotting

The total protein was extracted using RIPA buffer with phosphatase and protease inhibitors. After separation by SDS-PAGE, the protein was transferred to polyvinylidene fluoride membranes at 250 mA for 100 min. Five percent skim milk was used to block the membrane. Next, the membrane was incubated with primary antibodies (RPRD1B, Proteintech #24542-1-AP; β-Actin, Cell Signaling Technology #3700) at 4 °C for 12 h. Subsequently, the membrane was incubated with the secondary antibody at 37 °C for 60 min. After washing three times with TBST, the signal was detected by chemiluminescence.

### Colony formation assay

500 cells were seeded into six-well plates. After one week, cells were fixed and stained with crystal violet. The clone areas were measured using the ImageJ program and presented as mean ± standard deviation (SD) from three independent experiments.

### Cell proliferation assay

Cell viability was assessed with Cell Counting Kit-8 (Dojindo, Kumamoto, Japan) following the manufacturer’s protocol.

### Cell invasion assay

Cell invasion assay was performed using the Transwell chamber (Millipore Corp., Bedford, MA). A total of 5 × 10^4^ cells in 200 µL serum-free medium were added to the upper chamber coated with Matrigel (BD Biosciences, San Jose, CA, USA). At the same time, complete medium was added to the lower chamber. After 48 h, invaded cells were fixed in methanol and stained with 0.1% crystal violet. Photographs were taken and analyzed with Image J software to measure the invasion area.

### Animal assay

For the xenograft model, 5 × 10^6^ SW620 cells were subcutaneously injected into the right or left dorsal flank of each 4-week-old male Balb/c nude mouse, respectively. Tumor volume was measured weekly and calculated using the formula V = (W2 × L)/2, (V = volume, L = length, W = width). All mice were euthanized 6 weeks after injection, and the tumors were removed and weighed.

### Statistical analysis

A comparison of gene expression differences was conducted using the Wilcoxon Rank Sum Test and the Kruskal–Wallis Test. Spearman or Pearson correlation analysis was employed to determine the correlation between the two groups. Kaplan–Meier method and Cox regression analysis were used to compare survival characteristics. *P* < 0.05 (*), *P* < 0.01 (**), and *P* < 0.001 (***) were considered significant.

## Results

### RPRD1B expression is upregulated in multiple tumors

**Fig. 1:**
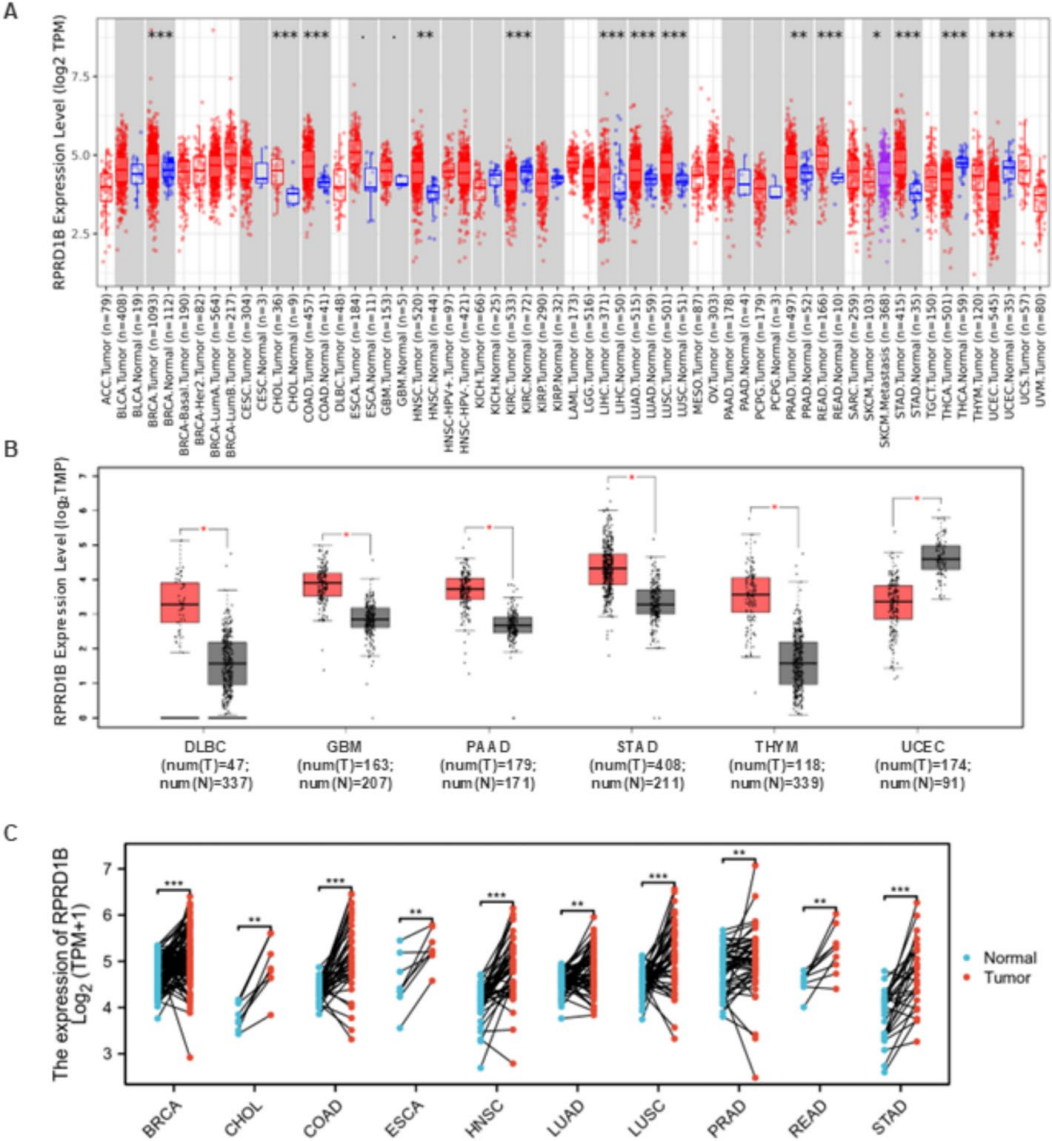
The mRNA expression profiles of RPRD1B across different studies combining data from The Cancer Genome Atlas (TCGA) and the Genotype-Tissue Expression (GTEx) project. **A** The expression level of RPRD1B in various cancers was analyzed using TIMER2.0. *P < 0.05; **P < 0.01; ***P < 0.001. **B** The RPRD1B expression profiles in different tumor types were examined with GEPIA2. Red indicates tumor groups, while blue indicates their corresponding normal controls. **C** For the six selected cancer types in the TCGA project, their matched normal tissues from the GTEx database were used as normal controls

The expression levels of RPRD1B in various cancer types were analyzed using data from The Cancer Genome Atlas (TCGA). RPRD1B expression was significantly higher in 10 tumor types, including breast cancer (BRCA), cholangiocarcinoma (CHOL), colon cancer (COAD), head and neck squamous cell carcinoma (HNSC), liver hepatocellular carcinoma (LIHC), lung adenocarcinoma (LUAD), lung squamous cell carcinoma (LUSC), prostate cancer (PRAD), rectal adenocarcinoma (READ) and stomach cancer (STAD). In contrast, RPRD1B was downregulated in kidney renal clear cell carcinoma (KIRC), skin cutaneous melanoma (SKCM), thyroid cancer (THCA), and uterine corpus endometrial carcinoma (UCEC) (Fig. 1A). Furthermore, based on GEO datasets, RPRD1B expression was upregulated in five tumors: diffuse large B-cell lymphoma (DLBC), glioblastoma (GBM), pancreatic adenocarcinoma (PAAD), STAD and thymoma (THYM), and downregulated in uterine corpus endometrial carcinoma (UCEC) (Fig. 1B). Since some tumors lacked corresponding normal tissues, we combined TCGA and GTEx data to study RPRD1B expression across 33 tumors and found that RPRD1B was upregulated in ten of these tumors compared to their normal tissues, including BRCA, CHOL, COAD, esophageal carcinoma (ESCA), HNSC, LUAD, LUSC, PRAD, READ, and STAD (Fig. 1C). The overall findings were consistent.

### Subcellular localization and protein expression of RPRD1B in pan‑cancer

**Fig. 2:**
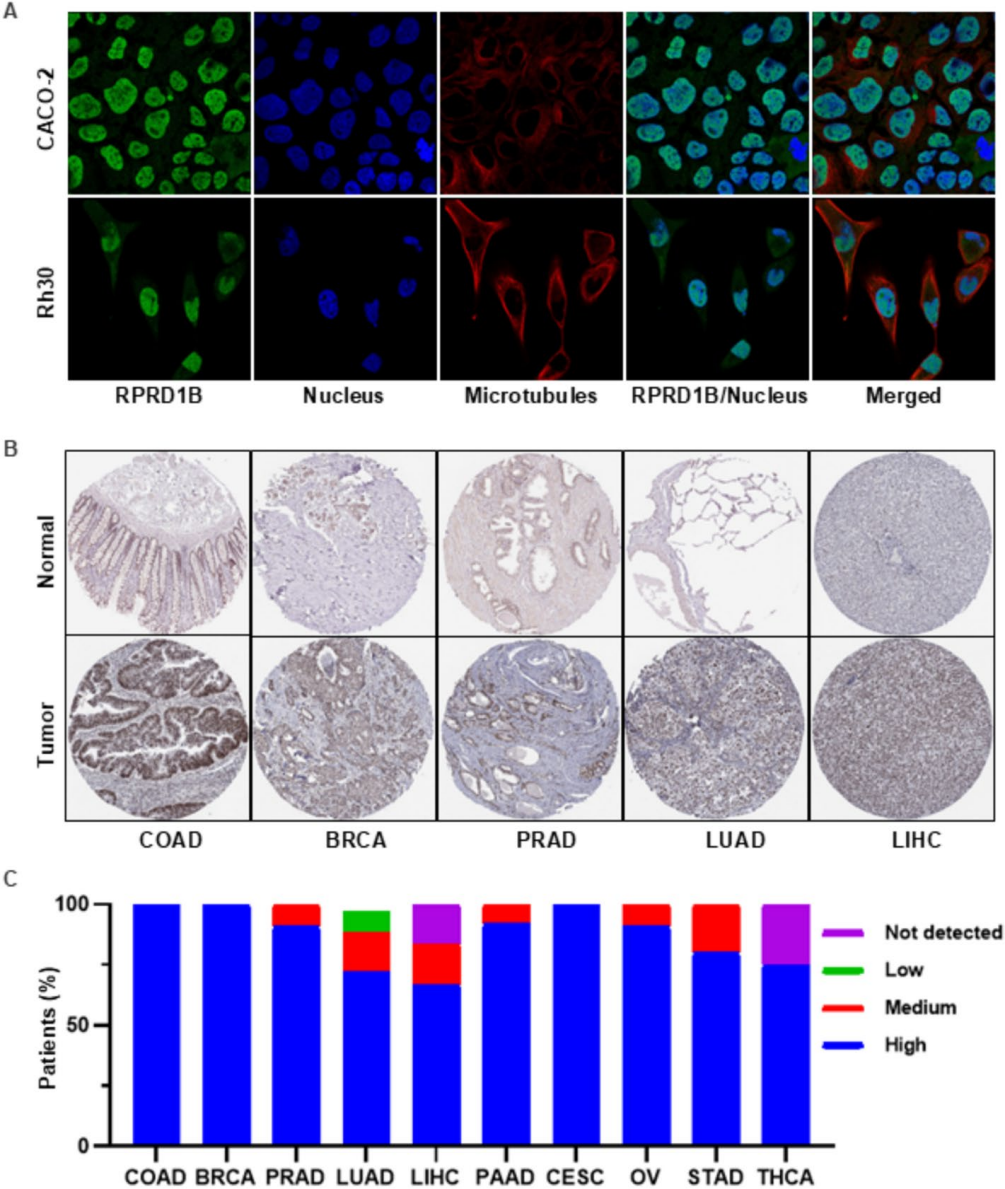
Subcellular localization and protein expression of RPRD1B. **A** Localization of RPRD1B within cells. **B** IHC results showing RPRD1B protein levels in various tumors from the HPA database. **C** Statistical analysis of RPRD1B protein expression across different tumor types in the HPA database. (COAD: n = 12, BRCA: n = 10, PRAD: n = 10, LUAD: n = 11, LIHC: n = 10, PAAD: n = 10, CESC: n = 11, OV: n = 12, STAD: n = 11, THCA: n = 3). Negative—not detected; weak < 25%—not detected; weak combined with 25%-75% or 75%—low; moderate < 25%—low; moderate combined with 25%-75% or 75%—medium; strong < 25%—medium; strong combined with 25%-75% or 75%—high

The subcellular localization of RPRD1B was determined from online data through immunofluorescence analysis of nuclei and microtubules in CACO-2 and Rh30 cells. The immunofluorescence results showed that RPRD1B was located in the nucleoplasm of both cell lines (Fig. 2A). These findings further support the universality of RPRD1B and its potential as a target.

The immunohistochemical staining from the Human Protein Atlas (HPA) database showed that RPRD1B staining is moderate in normal tissues, while it is significantly increased in tumor tissues (Fig. 2B). The results of immunohistochemistry indicated that RPRD1B has high expression in most tumor patients, especially in COAD, BRCA and cervical squamous cell carcinoma and endocervical adenocarcinoma (CESC). Moderate expression was found in a few cases, including PRAD, LUAD, LIHC, PAAD, ovarian serous cystadenocarcinoma (OV) and STAD, while undetectable expression was observed in a small number of patients, such as LIHC and thyroid carcinoma (THCA) (Fig. 2C). Overall, these findings show that RPRD1B is mostly highly expressed in tumor tissues.

### Amplification is the most common genetic alteration of RPRD1B

**Fig. 3:**
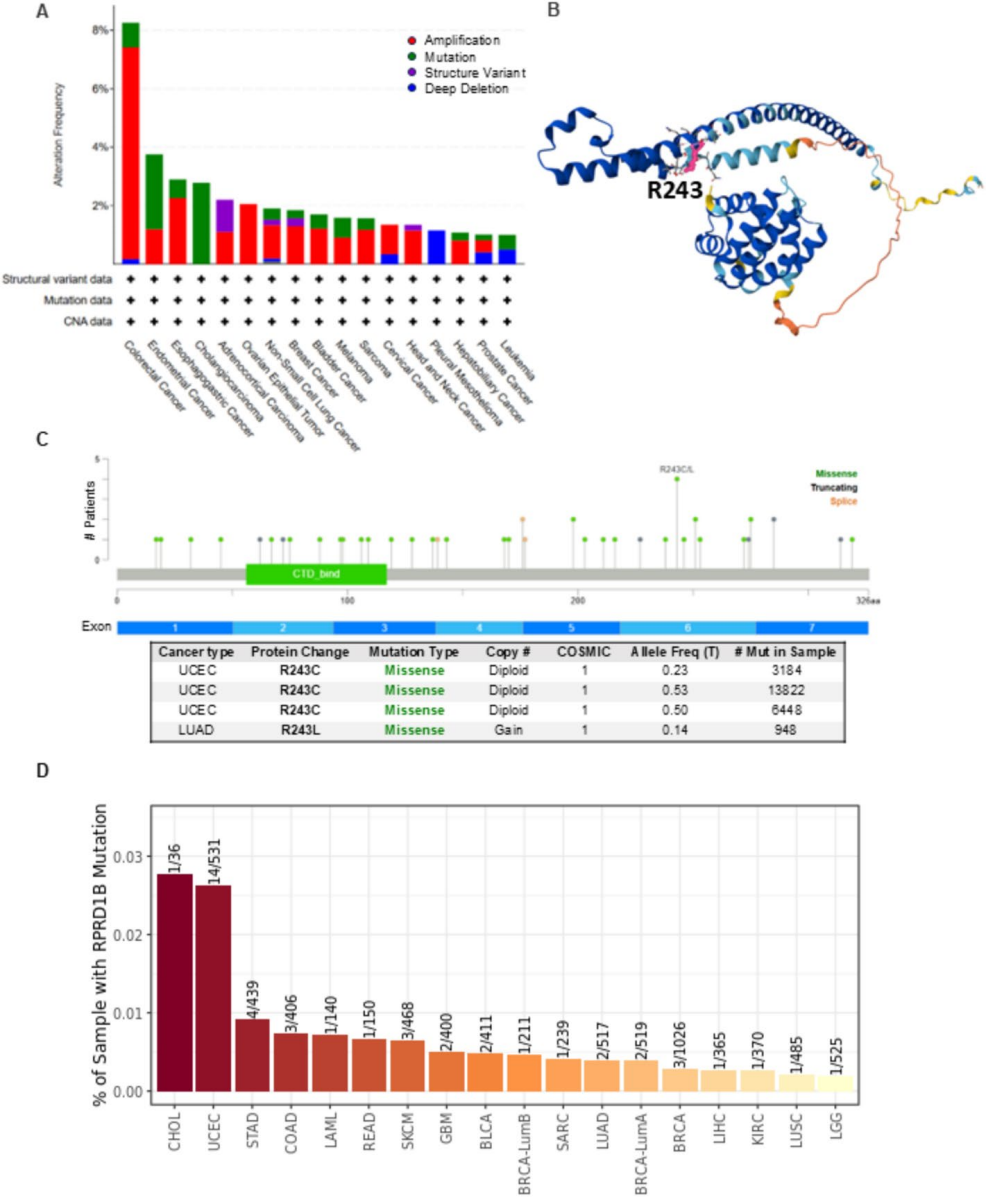
Genetic variation of RPRD1B in different types of tumors. **A** Genetic variation of RPRD1B in different types of tumors through the cBioPortal database. There are 32 studies with a total of 10,953 patients with 10,967 samples. **B** The crystal structure of RPRD1B was predicated in AlphaFold (Q9NQG5) and the position of the most common mutation R243 was marked in pink. **C** The most observed mutation was R243 in the TCGA cohort, which was predicted to be missense. **D** The mutation distribution in patients with diverse cancers

Genetic alterations of RPRD1B were examined across various cancer samples from TCGA cohorts. Our analysis revealed that gene amplification is the predominant genetic alteration affecting the RPRD1B locus in the TCGA pan-cancer dataset. These were mainly observed in COAD (7.24%), uterine corpus endometrial carcinoma (USEC) (0.95%), ESCA (2.2%), adrenocortical carcinoma (ACC) (1.1%), OV (2.05%), LUSC (1.64%), BRCA (1.29%), bladder urothelial carcinoma (BLCA) (1.22%), melanoma (0.9%), sarcoma (1.18%), CESC (1.01%), HNSC (1.15%), LIHC (0.81%) and PRAD (0.4%) patients (Fig. 3A).

The overall mutation rate of RPRD1B was relatively low (0.5%) across all cancer types. A total of 54 mutations were identified in the RPRD1B gene among TCGA tumor samples, consisting of 33 missenses, 4 nonsense mutations, 4 splice site alterations, 8 fusions, and 5 frameshift mutations. These mutations were spread throughout the RPRD1B gene, affecting both domain and non-domain regions (Fig. 3B). The most frequently observed mutations were R243C and R243L in the TCGA cohort, predicted to be inactivating (Fig. 3B, C). Additionally, we used the TIMER2.0 database to further examine RPRD1B gene mutations across various human cancers. The mutations were mostly found in patients with CHOL (1 out of 36) and UCEC (14 out of 531) (Fig. 3D).

### Enrichment pathways identified for RPRD1B

**Fig. 4:**
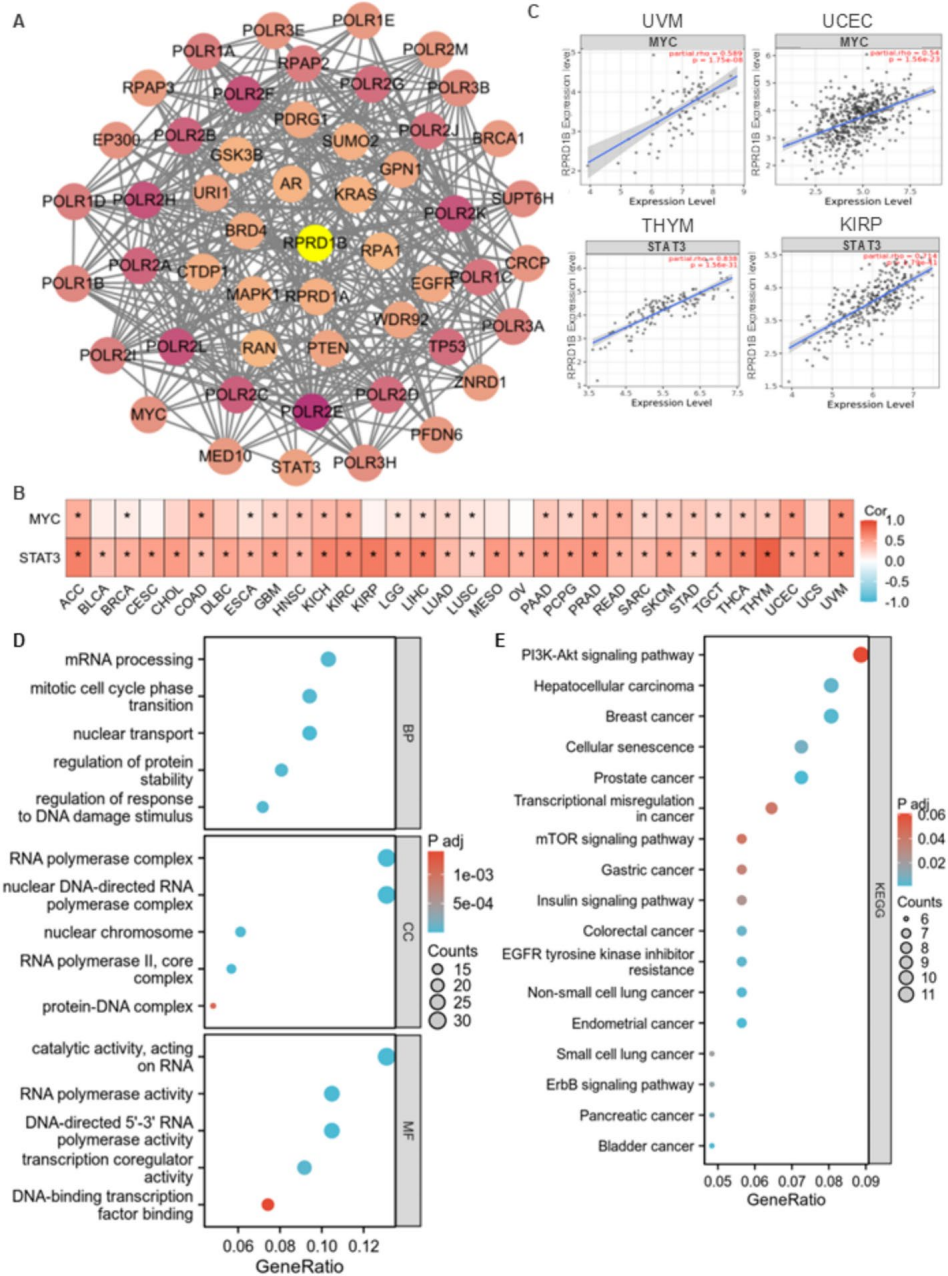
Co-expressed genes and enrichment analysis of RPRD1B. (A) The BioGRID analysis identified a total of 246 proteins that interact with RPRD1B, with some representative proteins shown in the figure. (B, C) The correlation between RPRD1B and MYC/STAT3 expression. (D) GO enrichment analysis of RPRD1B interactors. (E) KEGG pathway analysis of RPRD1B interactors

To explore the potential role of RPRD1B in tumorigenesis, this study identified genes associated with RPRD1B expression and those targeted by RPRD1B-binding proteins for pathway enrichment analysis. A total of 246 proteins were obtained through BioGRID analysis, forming an interaction network, with some representative proteins shown in Fig. 4A. Notably, tumor-associated transcription factors Myc and STAT3 interact with RPRD1B, respectively. Further analysis using the TIMER2.0 database revealed that Myc and STAT3 showed positive correlations with RPRD1B in most cancers (Fig. 4B). Specifically, Myc had the strongest interaction with RPRD1B in uveal melanoma (UVM) (rho = 0.59, P < 0.05) and UCEC (rho = 0.54, P < 0.05), while STAT3 exhibited the most robust interaction with RPRD1B in THYM (rho = 0.84, P < 0.05) and KIRP (rho = 0.71, P < 0.05) (Fig. 4C).

The datasets were combined for further KEGG and GO enrichment analysis. Biological process (BP) enrichment analysis indicated that RPRD1B-related genes mainly participate in mRNA processing, mitotic cell cycle transition and DNA damage response processes. In cellular component (CC) enrichment analysis, RPRD1B-related genes were primarily enriched in RNA polymerase complex and nuclear DNA-directed RNA polymerase complex. Molecular function (MF) analysis showed that RPRD1B's role is associated with catalytic activity on RNA and RNA polymerase activity (Fig. 4D). Additionally, KEGG pathway analysis revealed that RPRD1B-related genes are involved in the PI3K-Akt signaling pathway, mTOR signaling pathway and several cancer-related pathways, including hepatocellular carcinoma, breast cancer, gastric cancer, colorectal cancer, lung cancer, pancreatic cancer and bladder cancer (Fig. 4E).

### The potential association between RPRD1B and immune infiltration

**Fig. 5:**
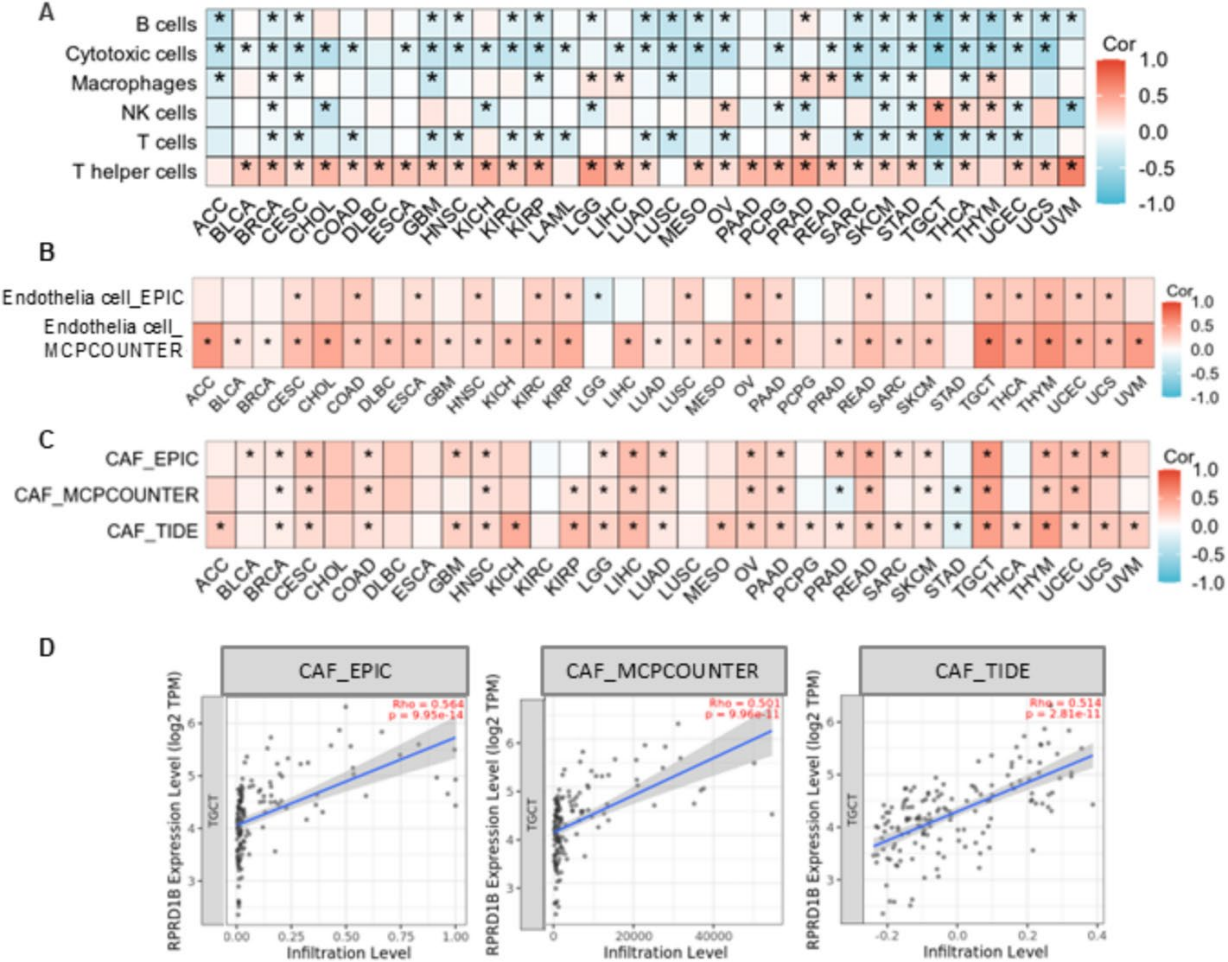
The possible link between RPRD1B expression and immune infiltration. **A** The ssGSEA algorithm was used to examine the relationship between RPRD1B expression and immune cell infiltration across different tumors. **B** Two algorithms were employed to investigate the relationship between RPRD1B expression and endothelium cell infiltration in various tumors. **C**, **D** Three algorithms were used to analyze the relationship between RPRD1B expression and CAFs infiltration in different cancers. The correlation and scatterplot for TGCT are shown in figure D. CAF, Cancer-associated fibroblast. *P < 0.05

The algorithm of ssGSEA (single sample gene set enrichment analysis) was used to explore the correlations between immune cell infiltration levels and RPRD1B expression across different cancer types. RPRD1B expression showed a significant positive correlation with the infiltration level of T helper cells in most tumor types (Fig. 5A). A significant positive correlation was also observed between RPRD1B expression and endothelial cells (Fig. 5B), as well as CAFs (cancer-associated fibroblasts) (Fig. 5C) in most tumor types. The strongest correlation between RPRD1B and CAFs was seen in testicular germ cell tumors (TGCT) (Fig. 5C), and this finding was further confirmed using the TIMER2.0 database. The EPIC, MCP-counter, and TIDE algorithms analyzed that the correlation coefficients between RPRD1B and CAFs were 0.56, 0.50 and 0.51, respectively (Fig. 5D).

### Relationship between RPRD1B and genomic heterogeneity

**Fig. 6:**
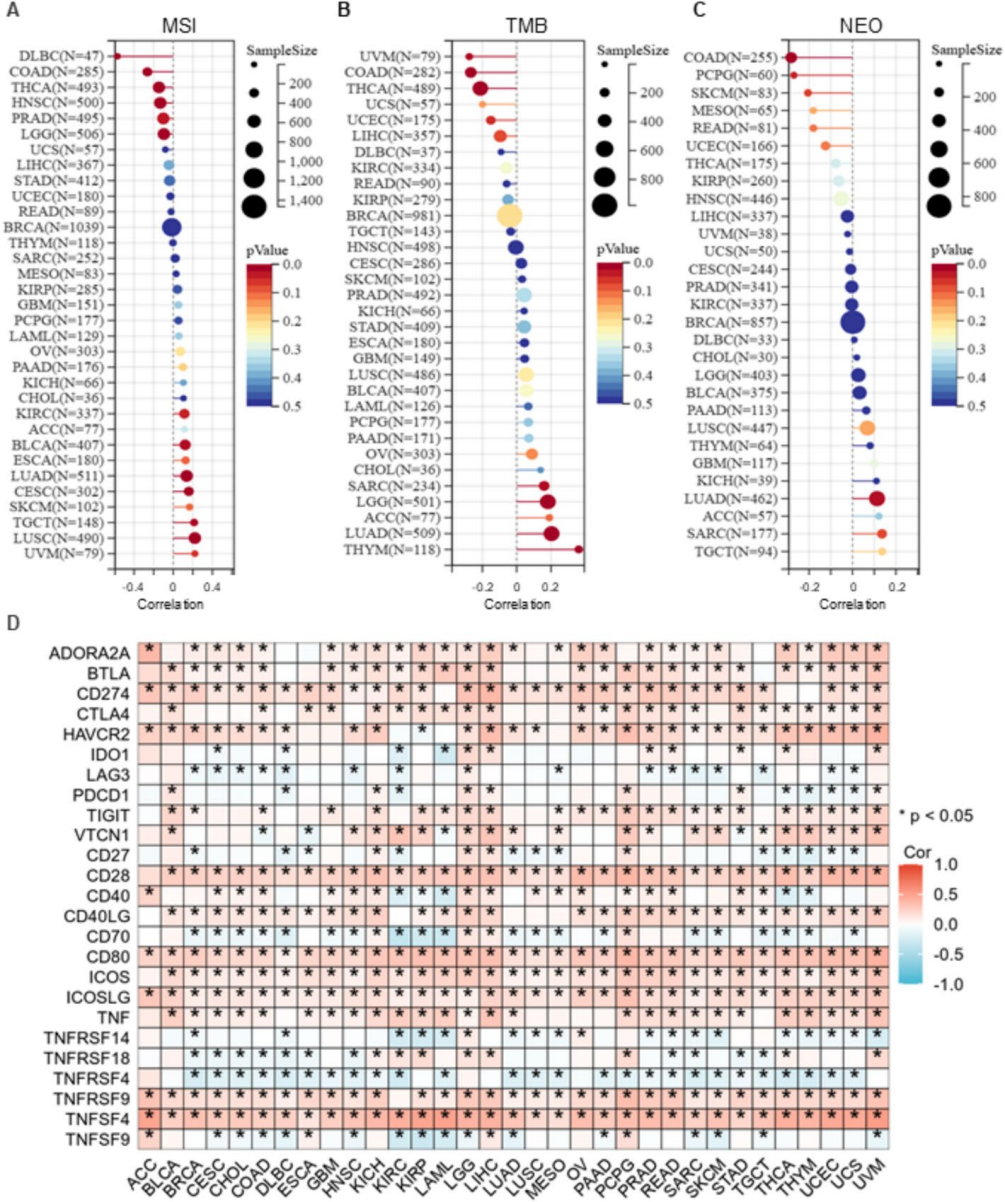
Correlation analysis between RPRD1B and tumor immunity markers. The relationship between RPRD1B mRNA expression and MSI (**A**), TMB (**B**), neoantigens (**C**) and ICP-gene (**D**) across various cancers

Antitumor immunity is a crucial factor influencing response to immunotherapy and is closely linked to microsatellite instability (MSI), tumor mutation burden (TMB), and tumor-specific neoantigens [24]. MSI-high (MSI-H) and high-TMB tumors generally show increased sensitivity to immune checkpoint blockade [25]. Neoantigens, which originate from somatic mutations and are uniquely expressed by tumor cells, serve as key targets for T cell-mediated antitumor immune responses [26]. Therefore, we examined the relationship between RPRD1B expression and MSI, TMB, and neoantigen levels across various cancers to evaluate its potential as a predictor of immunotherapy response. RPRD1B expression demonstrated a positive correlation with MSI in BLCA (P = 0.0014), CESC (P = 0.0056), KIRC (P = 0.037), LUAD (P = 0.002), LUSC (P = 8.32e-07) and TGCT (P = 0.009), while showing a negative correlation in COAD (P = 6.46e-06), READ (P = 1.70e-06), DLBC (P = 2.82e-05), brain lower grade glioma (LGG) (P = 0.03), PRAD (P = 0.02) and THCA (P = 0.001) (Fig. 6A). Similarly, RPRD1B expression was positively associated with TMB in LGG (P = 4.37e-05), LUAD (P = 3.98e-06), SARC (P = 0.014) and THYM (P = 5.62e-05), but negatively related to TMB in COAD (P = 5.13e-06), READ (P = 3.24e-07), LIHC (P = 0.04), THCA (P = 5.50e-06), UCEC (P = 4.57e-02) and UVM (P = 0.014) (Fig. 6B). Furthermore, RPRD1B expression also showed a positive correlation with neoantigens in LUAD (P = 0.034), while it was inversely related to neoantigens in COAD (P = 5.75e-05), READ (P = 4.27e-07), KICH (P = 0.034) and UCEC (P = 0.007) (Fig. 6C).

Immune checkpoint (ICP) blockade proteins are essential targets for cancer immunotherapy because they regulate immune cell infiltration within the tumor microenvironment [27]. Our analysis of the relationship between RPRD1B expression and 25 ICP genes across various cancer types showed widespread positive correlations. Notably, strong associations were seen in LGG (23/25 genes), LIHC (22/25), KICH (20/25), and PCPG (20/25) (Fig. 6D). This suggests that RPRD1B may play a role in increasing tumor sensitivity to ICP blockade therapies in these cancers.

### Prognostic implications of RPRD1B in pan-cancer

**Fig. 7:**
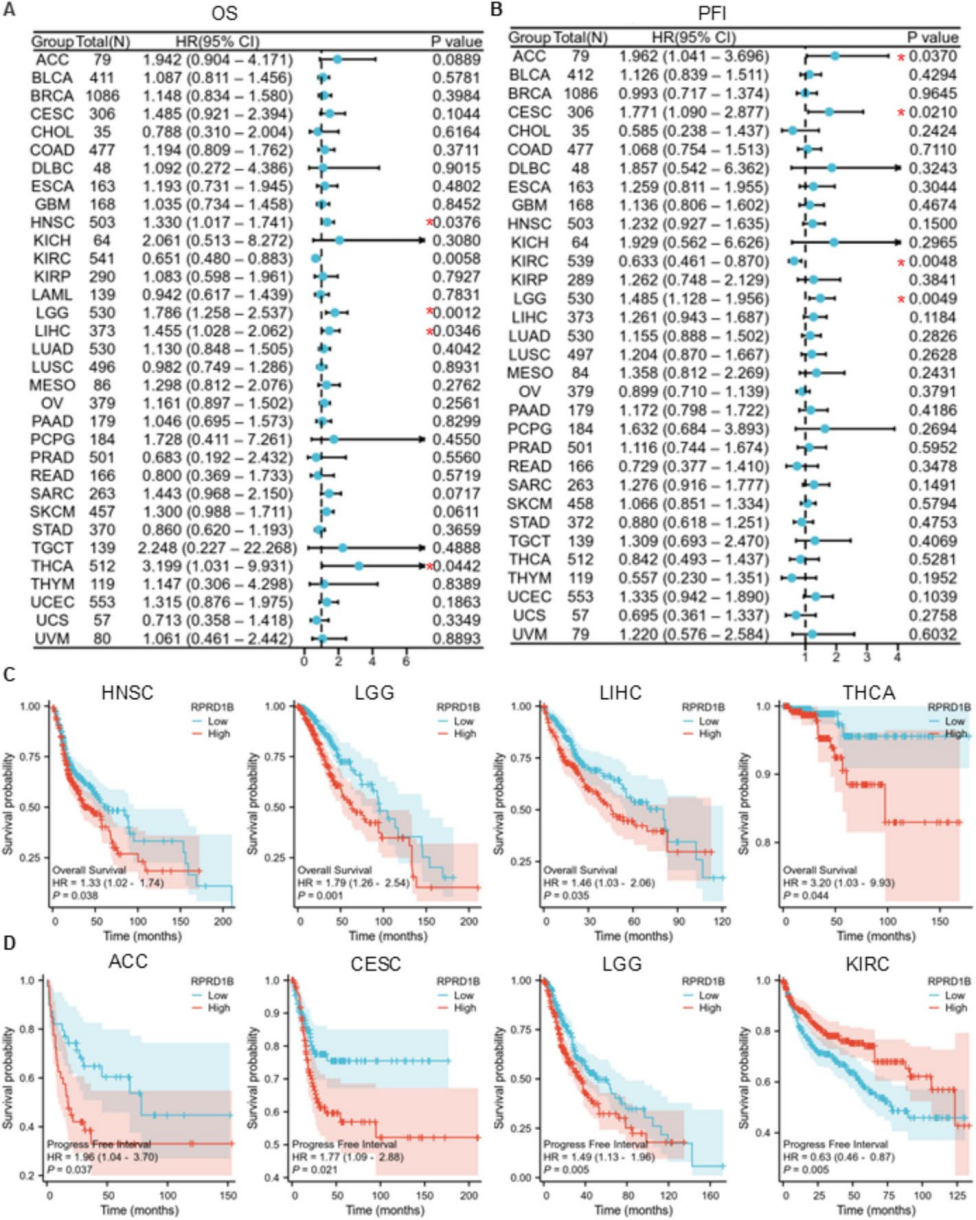
The impact of RPRD1B expression on OS and PFI. **A**, **B** Cox proportional hazards model demonstrating the association between RPRD1B and survival outcomes, including OS (**A**) and PFI (**B**). **C**, **D** Kaplan–Meier survival curves used to analyze the correlation between RPRD1B expression and OS (**C**) and PFI (**D**) in patients with different tumor types

To explore the potential role of RPRD1B in cancer prognosis, we conducted survival analyses including OS (overall survival) and PFI (progress free interval) across various cancer types. Cox proportional hazards model analysis showed that higher RPRD1B expression was significantly linked to poorer OS in HNSC, LGG, LIHC, and THCA, suggesting its role as a high-risk factor (Fig. 7A). At the same time, increased RPRD1B expression was also significantly associated with reduced PFI in ACC, CESC and LGG, further supporting its high-risk prognostic significance (Fig. 7B). However, in KIRC patients, it indicated a better PFI (Fig. 7B).

Kaplan–Meier (KM) survival curves comparing RPRD1B high and low expressing patients were also constructed to further assess the prognostic potential of RPRD1B. The results showed that high RPRD1B expression predicted poorer OS in HNSC, LGG, LIHC, and THCA (Fig. 7C). Concerning PFI, high RPRD1B expression was associated with worse outcomes in ACC, CESC and LGG, but unexpectedly showed a protective association in KIRC (Fig. 7D). These findings suggest that RPRD1B could serve as an independent prognostic biomarker for these cancers.

### Diagnostic value of RPRD1B in pan-cancer

**Fig. 8:**
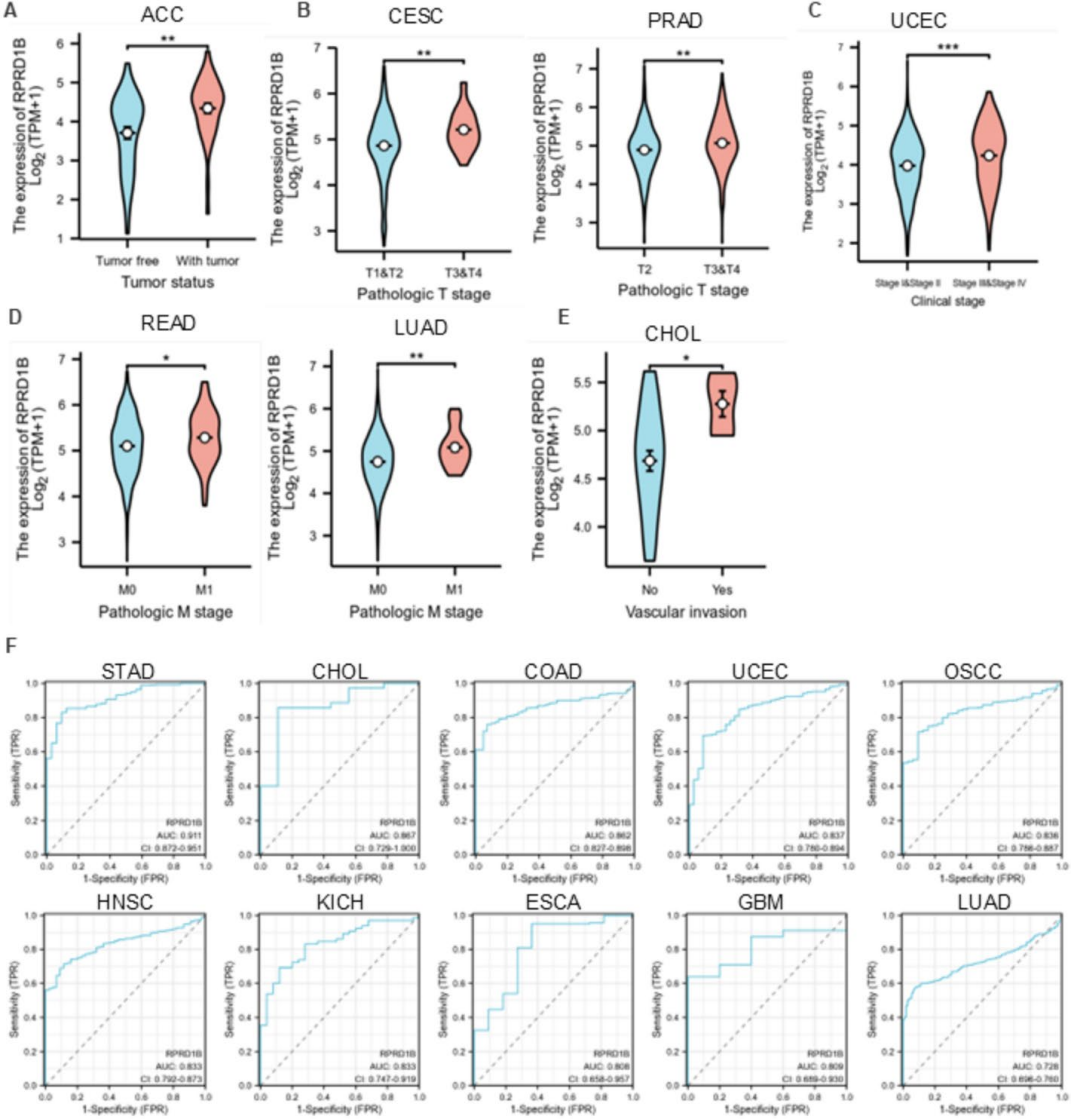
The diagnostic value of RPRD1B expression in various tumor types. **A**–**E** Correlation between RPRD1B expression and clinicopathological features. ***P < 0.001, **P < 0.01, *P < 0.05. **F** ROC curves of RPRD1B for assessing different tumor types

To examine the clinical significance of RPRD1B, this study explored the relationship between RPRD1B expression and clinicopathological features. In ACC, RPRD1B levels were significantly higher in patients with tumors compared to those without (Fig. 8A). In CESC and PRAD, RPRD1B expression was significantly elevated in patients with later pathologic T stages (T3&T4) compared to earlier stages (T1&T2) (Fig. 8B). For UCEC, RPRD1B levels were notably higher in stages III & IV than in stages I & II (Fig. 8C). In READ and LUAD, RPRD1B expression was increased in patients with M1 stage (distant metastasis present) compared to M0 (no distant metastasis) (Fig. 8D). In CHOL, RPRD1B expression was higher among patients with vascular invasion than those without (Fig. 8E). These findings indicate that RPRD1B expression correlates with various clinicopathological features across different cancer types.

The receiver operator characteristic (ROC) curve was further used to analyze the diagnostic potential of RPRD1B across various cancer types. The analysis showed that RPRD1B had good diagnostic value in 10 tumor types (AUC > 0.7). Specifically, it demonstrated strong diagnostic capability in the following cancers, with AUC values ranging from 0.8 to 0.9: CHOL, COAD, UCEC, oral squamous cell carcinoma (OSCC), HNSC, KICH, ESCA and GBM. Additionally, RPRD1B showed excellent diagnostic ability in STAD, with an AUC of 0.911 (Fig. 8F).

### Knockdown of RPRD1B suppressed proliferation, invasion and tumor growth of COAD

**Fig. 9:**
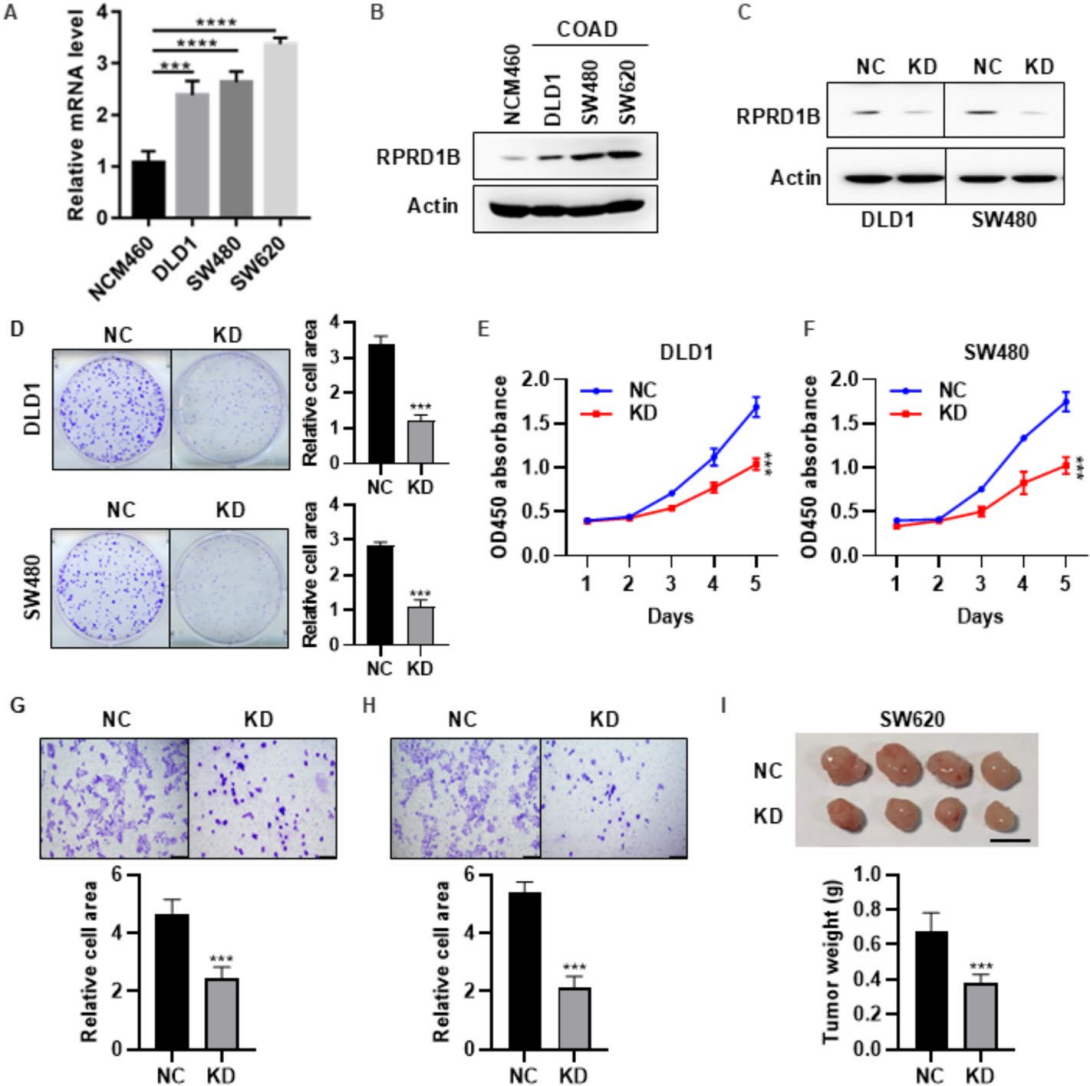
Knockdown of RPRD1B suppresses proliferation, invasion and tumor growth in COAD. **A** The mRNA expression levels of RPRD1B in NCM460, DLD1, SW480, and SW620 cells were measured by RT-qPCR. **B** Western blot analysis was used to detect RPRD1B protein expression in these cells. **C** RPRD1B knockdown (KD) efficiency was detected by western blot. **D** Colony formation assays assessed the proliferation and colony formation of DLD1 and SW480 cells following RPRD1B knockdown. **E**, **F** CCK-8 reagent was used to measure the proliferation of DLD1 (**E**) and SW480 (**F**) cells after RPRD1B knockdown. **G**, **H** Transwell invasion assays evaluated the migration and invasion capabilities of DLD1 (**G**) and SW480 (**H**) cells following RPRD1B knockdown. Scale bar = 100 μm. **I** Tumor growth was monitored in xenograft models generated with SW620 cells after RPRD1B knockdown. Scale bar = 1 cm.***P < 0.001

We examined the expression of RPRD1B in COAD, and the results revealed that the mRNA and protein levels of RPRD1B in tuomor cell lines were significantly higher than those in the normal colonic epithelial cell NCM460 (Fig. 9A, B). To explore the impact of RPRD1B on COAD cell functions, we conducted RPRD1B knockdown cell lines (Fig. 9C). Colony formation assay (Fig. 9D), proliferation assay (Fig. 9E, F), and Transwell assay (Fig. 9G, H) results showed that RPRD1B knockdown suppressed the cell proliferation and invasion of DLD1 and SW480 cells. Furthermore, tumor xenograft experiments showed that RPRD1B knockdown suppressed the growth of SW620 cells in vivo (Fig. 9I). These results demonstrated that RPRD1B promotes the proliferation and invasion of COAD cells both in vitro and in vivo.

## Discussion

Previous research indicates that RPRD1B expression is linked to tumor progression and patient survival, highlighting its role in cancer biology. This study combines multi-omics data to thoroughly examine RPRD1B’s expression profile, prognostic value, diagnostic ability, and its connections to genetic alterations, the tumor immune microenvironment, and key signaling pathways across various cancers. These findings offer new insights into its biological function and clinical importance for patient outcomes. The analysis revealed that RPRD1B is broadly upregulated in 10 tumor types, implying it has a significant oncogenic role in tumor development and progression. This supports earlier findings that associate RPRD1B’s expression with poorer survival outcomes [28–30], reinforcing its potential as a prognostic marker in clinical practice. Additionally, the link between RPRD1B expression and certain cancer types suggests that its function may differ depending on the tumor’s microenvironment and genetic background. The study also identified missense mutations in RPRD1B among UCEC patients and high-frequency amplification mutations in other tumor types, such as COAD and OV. These factors contribute to elevated RPRD1B levels and are associated with worse prognosis.

The comprehensive analysis of genetic alterations offers important insights into how RPRD1B promotes cancer. The frequent amplification of the RPRD1B gene across various cancers, especially in COAD, UCEC, and OV, is a key genetic mechanism behind its widespread overexpression. This pattern indicates that cancer cells often increase gene copies to boost RPRD1B levels, giving them a growth advantage. As a result, tumors with RPRD1B amplification may rely on this gene, making RPRD1B a promising target for therapy. In contrast, the low overall mutation rate suggests that RPRD1B is not typically a gene that is mutated often in cancer. However, the recurring missense mutations (e.g., R243C/L), though rare, imply there might be groups of patients where mutant RPRD1B proteins have altered functions. The clustering of these mutations and their predicted damaging effects should be studied further to see if they define a separate biological subtype or influence how patients respond to treatment.

The results of survival analysis showed that, among various tumor types, patients with high RPRD1B expression had a significantly lower overall survival rate compared to those with low expression levels. This finding highlights the potential of RPRD1B as a tool for stratifying patients in clinical practice, helping to identify high-risk individuals who might benefit from more aggressive treatment. Additionally, the study's analysis indicates that RPRD1B's prognostic value remains consistent across different populations, although further research is needed to determine its relevance across different races and genders.

Additionally, correlation analysis showed a significant relationship between RPRD1B and other key oncogenes or tumor suppressor genes, such as Myc, KRAS, STAT3, EGFR, TP53, AR, BRCA1, and GSK3β. This suggests that RPRD1B may be closely involved in critical signaling pathways linked to cancer progression. Existing studies have demonstrated that RPRD1B interacts with important carcinogens like STAT3 [12] and Myc [31], highlighting its crucial role in carcinogenic signal transduction and its potential as a therapeutic target. Based on these new findings, we speculate that RPRD1B might also have functional connections with other significant regulatory factors. This hypothesis deserves further investigation in future studies.

Currently, the specific role of RPRD1B in the tumor immune microenvironment (TIME) remains unclear. Our multi-omics approach, leveraging tools like TIMER2.0, aligns with the paradigm of integrative TME deconvolution as exemplified by the immuno-oncology biological research (IOBR) package [32]. The findings of this study show that high RPRD1B expression is significantly linked to changes in immune cell infiltration, especially with T helper cell levels across 19 cancer types, indicating its possible role in immune regulation. This broad association suggests that RPRD1B may influence a key regulatory point within the TME, similar to the critical transition states (for example, Stage II as a critical transition signal) identified in thyroid cancer through dynamic network biomarker (DNB) analysis [33].

Additionally, RPRD1B expression was linked to the infiltration of endothelial cells and CAFs, indicating it may influence angiogenesis [34], fibroblast activation [35], stromal remodeling [36] and stromal-immune crosstalk [37]. The positive correlation between RPRD1B and CAFs in tumors like TGCT is especially intriguing. Tools like IOBR are specifically designed to analyze such ligand-receptor interactions and cellular crosstalk within the TME [32], and future studies using these methods could clearly define the signaling pathways through which RPRD1B affects fibroblast recruitment and activation.

Notably, RPRD1B shows broad associations with key tumor immunity markers like CTLA-4, highlighting its potential as a therapeutic target to enhance immunotherapy outcomes. The ability of RPRD1B to predict immunotherapy response may vary depending on the overall immune environment of the tumor. This complexity emphasizes the importance of advanced predictive models that combine multi-omics features, such as the integrated Machine Learning and Genetic Algorithm-driven Multiomics analysis (iMLGAM) framework [38]. iMLGAM illustrates that sophisticated computational integration of diverse features can produce strong predictors of ICB response, surpassing single biomarkers. Our finding that RPRD1B expression correlates with TMB, MSI, and neoantigens in a cancer-type-dependent way further underscores this complexity. Future research could investigate whether adding RPRD1B to a broader multi-omics model, like iMLGAM, improves the prediction of immunotherapy benefits in specific cancers where RPRD1B is overexpressed. Additional studies on RPRD1B-targeted strategies could also boost responses to immune checkpoint blockade [39].

Mechanistically, RPRD1B appears to influence cellular proliferation by regulating genes involved in the cell cycle and transcription, as demonstrated by GO enrichment analysis. This aligns with previous research showing that RPRD1B directly interacts with RNA polymerase II to control cell cycle genes and promote cancer progression [10, 37, 38]. Additionally, KEGG pathway analysis identified key connections between RPRD1B and several cancer-related signaling pathways, supporting its potential as both a prognostic biomarker and a therapeutic target. Notably, RPRD1B also plays a role in regulating the PI3K-Akt signaling pathway and cellular senescence. Future studies should focus on experimentally confirming the specific mechanisms through which RPRD1B functions, which could aid in developing new targeted therapies, especially for cancers with RPRD1B overexpression. Furthermore, our GO enrichment analysis revealed that RPRD1B-associated genes are significantly involved in DNA damage response processes. This bioinformatic finding aligns with established molecular studies indicating that RPRD1B (also known as Kub5-Hera/KNH) forms a complex with Ku70 to participate in the non-homologous end joining (NHEJ) pathway of DNA double-strand break repair [13]. The dual role of RPRD1B in regulating both transcription and DNA repair offers a compelling mechanism for its strong oncogenic potential. On one hand, by enhancing the transcription of cell-cycle genes, it drives proliferation. On the other hand, by contributing to DNA repair, it may help cancer cells survive genotoxic stress, including that induced by chemotherapy or radiotherapy. This ability to promote proliferation while maintaining genomic stability could be a key factor in tumor progression and therapy resistance, highlighting RPRD1B as a particularly promising therapeutic target.

Furthermore, we confirmed the high expression of RPRD1B in COAD cell lines. By establishing RPRD1B knockdown models and conducting cellular functional assays, we verified that RPRD1B promotes the proliferation and invasion capabilities of COAD cells. The mouse model showed that knocking down RPRD1B significantly suppressed tumor growth in vivo. These experimental findings validate the reliability of our bioinformatics analysis and lay the groundwork for future studies of RPRD1B in tumors.

In conclusion, this research highlights the important role of RPRD1B as a potential prognostic biomarker across various cancer types, demonstrating its association with poor outcomes and its involvement in tumor biology. The findings underscore the need for further investigation into RPRD1B’s mechanistic pathways and its implications for personalized treatment strategies. By confirming RPRD1B’s significance in cancer prognosis, this work supports future efforts to translate these findings into clinical practice, potentially guiding more tailored treatment decisions and improving patient care.

## Conclusion

This study offers a detailed analysis of the genetic, immune, and clinical aspects related to RPRD1B in pan-cancer. Our results show that RPRD1B is overexpressed in many tumor types and is strongly linked to cancer-related pathways. High levels of RPRD1B are associated with the infiltration of immune cells, endothelial cells, and CAFs, as well as tumor immune markers in specific cancers, highlighting its role in remodeling the tumor microenvironment. Additionally, RPRD1B expression correlates with poorer clinical outcomes, indicating its potential as a prognostic marker. Elevated RPRD1B levels also have predictive value for diagnosing and forecasting certain cancers. Our findings provide new insights into RPRD1B’s role in cancer and suggest it could be a promising therapeutic target. Future research should investigate RPRD1B across more tumor types and clarify the mechanisms driving its impact on cancer progression.

## Data Availability

Data supporting the findings of this study were obtained from publicly accessible websites as detailed in the “Methods” section. Further inquiries can be directed to the corresponding author upon reasonable request.
